# Oil Yield and Bioactive Compounds of *Moringa oleifera* Trees Grown Under Saline Conditions

**DOI:** 10.3390/plants14040509

**Published:** 2025-02-07

**Authors:** Hala M. Bayomy, Eman S. Alamri, Basmah M. Alharbi, Seham E. Almasoudi, Nawal A. Ozaybi, Ghena M. Mohammed, Esmail A. Genaidy, Amira K. G. Atteya

**Affiliations:** 1 Food Science and Nutrition Department, Faculty of Science, University of Tabuk, Tabuk 71491, Saudi Arabia; 2 Biology Department, Faculty of Science, University of Tabuk, Tabuk 71491, Saudi Arabia; 3 Pomology Department, Agricultural and Biology Research Institute, National Research Centre, Giza 12622, Egypt; 4 Horticulture Department, Damanhour University, Damanhour 22516, Egypt

**Keywords:** moringa oil, seaweed *Ulva lactuca*, bioactive compound, antioxidant activity

## Abstract

*Moringa oleifera* is a tree with various applications. Desertification and salinity are major constraints to crop productivity worldwide, especially in Saudi Arabia. Therefore, it is essential that plants alleviate and adapt to salt stress. Many physiological, pharmacological, and molecular strategies are employed by plants to lessen the effects of salinity stress. In this work, plants were grown under different salinity levels and treated with a foliar spray of seaweed extract to evaluate the fixed oil using GC/MS analysis, free proline and total soluble proteins using colorimetric methods, total phenolic content using Folin–Ciocalteu phenol reagent, total flavonoids using a spectrophotometric method, and antioxidant activity using the DPPH method. The study has shown that applying seaweed extract to plants grown under different salinity conditions improves seed oil yield, proline levels, soluble proteins, phenolic content, flavonoids, and antioxidant activity. As salinity increases, the oil yield decreases, but the levels of proline, phenols, flavonoids, and antioxidant activity rise. Seaweed extract application also reduces protein breakdown and boosts osmoprotectants. Salt stress decreases unsaturated fatty acids like oleic acid and increases saturated fatty acids like stearic acid. Overall, seaweed extract helps mitigate the adverse effects of salinity, enhancing oil yield and stress resistance in moringa trees.

## 1. Introduction

The *Moringa oleifera* tree grows to a small to medium size. Sometimes called the drumstick or horseradish tree, all of its parts are beneficial. The ten to fourteen species that comprise the single genus *Moringa* are found in the family Moringaceae. Among them, *M. oleifera* is the most commonly known. Although it originated in Northwest India, it is now widely grown throughout the tropical and subtropical regions of Central Asia, America, and Africa, including the Philippines, Thailand, Malaysia, and Pakistan [1,2]. In Saudi Arabia, it is also grown in private gardens and small plots. The *M. oleifera* tree has a high vitamin and mineral content [3]. Recently, its seeds have attracted interest as a source of plant oil due to their high oleic fatty acid content. This yellow-brown oil smells like almonds and is semi-solid [4]. As a substitute for polyunsaturated vegetable oils, oils high in oleic acid are becoming increasingly popular. Research indicates that these oils effectively preserve their oxidative stability during frying [5]. The *M. oleifera* tree has many beneficial applications, such as in animal feed, alley cropping, household cleaners, biogas, healthcare, organic fertilizer, decorative plants, gum, and water purification [6], because its oil can be used for both commercial and human consumption. In addition, a variety of illnesses, including skin infections, lung conditions, and high blood pressure, can be treated with the inflorescences, leaves, roots, and seeds of the *M. oleifera* tree [7].

Salinity stress is a major limiting factor for global crop production, negatively impacting plant growth and productivity [8]. It causes significant agricultural losses worldwide, affecting over 20% of arable land and 7% of the total land. If current trends persist, 50% of cultivable land may disappear by 2050 due to soil salinization [9]. The side effects of this problem, besides desertification, land degradation, and a declining precipitation rate, increasingly limit crop cultivation in arid and semiarid climate zones, such as Saudi Arabia [10]. Therefore, enhancing crops’ salt tolerance is vital for sustainable farming. Salinity disrupts water and nutrient absorption, reduces photosynthesis and transpiration, and impedes various metabolic processes. It also leads to the production of reactive oxygen species (ROS), contributing to oxidative stress [11,12]. To combat this, plants activate defense mechanisms like antioxidants (flavonoids and phenolics) and compatible solutes (proline and soluble proteins) to reduce osmotic stress and improve water absorption [13,14,15,16,17]. Plant biostimulants are commonly used in horticulture as a means of increasing stress tolerance and yield quality, as well as quantity. Plant biostimulants can efficiently promote the synthesis of osmolytes, which helps the plant resist the negative effects of salt stress and thereby reduces production losses [18]. Seaweed extract-derived plant biostimulants contain plant hormones such as auxins, cytokinins, and other hormone-like compounds [19,20]. Additionally, alginate, carrageenan, and fucoidan, which are found in seaweed extracts, are complex polysaccharides and phycocolloids that have been demonstrated to improve plant salinity tolerance [21,22,23]. Numerous studies have documented the advantages of applying seaweed extracts, which include improvements in yield parameters and secondary products [24,25,26]. Along with improving photosynthetic activity, seaweed extracts have also been shown to considerably increase the total chlorophyll content in plants by influencing the transpiration rate, stomatal conductance, and antioxidant activity [27,28]. These findings were discovered to have a substantial and favorable correlation with an increase in fruit, seed, and oil yield [29,30]. Thus, the primary objectives of this inquiry were to study the impact of salinity, seaweed extract, and their interaction on the *M. oleifera* tree’s oil yield and accumulation of secondary metabolites (such as proline and antioxidant compounds) and/or activating metabolic pathways through lessening the negative effects of salinization and improving plant growth.

## 2. Materials and Methods

### 2.1. Experimental Conditions and Design

In Tabuk, Saudi Arabia, during the 2021–2022 and 2022–2023 seasons, a pot experiment was conducted outside on the main campus of the University of Tabuk. The physical and chemical properties of soil and irrigation water were analyzed before starting the treatments according to the methods of Cottenie [31] and Jackson [32], and the results are shown in Table 1 and Table 2, respectively. There was open space in the field used for cultivation. Within randomized complete block designs, three replicate split plot designs were used. Two concentrations of seaweed foliar spray and five irrigation water salinity levels were used (Table 3). Three *M. oleifera* seeds were manually inserted into plastic pots measuring 30 cm in diameter and 20 cm in height in February 2021 and 2022. Each pot contained aproxmatly 13.44 cm^3^ of soil. Two weeks later, each pot contained a single living plant. Three hundred pots containing three hundred plants were distributed in 30 experimental units, ten plants for each replication per each treatment, respectively.

### 2.2. Plant Material

A single tree situated in a remote area of the study site yielded seeds belonging to *M. oleifera*. The seed for this tree was previously provided by Egypt’s National Research Center.

### 2.3. Applications of Irrigation

A tap water irrigation system was used to water the *M. oleifera* seeds. Thirty-three days after the date of sowing, the seedlings were treated with salt after fifty days of irrigation. After that, we watered them once or twice a week. Twice a week was the average frequency of irrigation with tap water or the salinity treatments (Table 3). To avoid salt buildup, the pot soil received one irrigation with tap water following each of the four saltwater irrigations. A pot of irrigation required about 750 mL of water up to the field’s capacity.

### 2.4. Seaweed Treatments

Seaweed (*Ulva lactuca*) was sourced from the Red Sea beaches of Alkhuraybah and Gayal. The seaweed was dried for 72 h at 70 °C to create *U. lactuca* aqueous extract solutions. After being dried and ground, a 40-mesh apparatus filtered the product. To create the extracts, 400 g of each ground plant material was macerated in 1000 mL of distilled water [33]. The solutions were mixed at room temperature in an orbital shaker for a full day. The extracts were filtered through Whatman filter paper No. 1. Only by diluting the dried extracts could the desired concentrations be attained. Algae extracts were evaluated for amino acids and lipids using AOAC’s [34] No. 984.13 and 2003.05 standards, while the minerals were assessed after digestion using Atomic Absorption Spectroscopy (Perkin-Elmer Model 2380 manufacturing, Waltham, MA, USA), according to the technique given by Bharathi et al. [35]. The results of numerous analyses conducted for the *U. lactuca* seaweed extract study are shown in Table 4. Every plant received a foliar spray containing a seaweed extract solution every 15 days until the pods were torn (approximately 15 months after planting) and fell off at a true two-leaf growth stage (approximately 9 days after sowing). The spray was applied manually using a sprayer. The control plants were sprayed with a similar amount of distilled water. During the spraying process, plastic covered the soil in the pot.

### 2.5. Measurements

All following studied measurements were examined at the end of the experiment. Five plants were randomly chosen from each block and fifteen plants from each treatment.

#### 2.5.1. Fixed Seed Oil Content

The fixed oil content per plant was computed using the estimation of the fixed oil percentage, in accordance with the method described by [36].

#### 2.5.2. GC/MS Analysis of Fixed Oil

After characterizing methanolic sulfuric acid using gas chromatography-mass spectrometry, fatty acid methyl esters were prepared. The fixed oil gas chromatography-mass spectrometry (GC/MS) analyses adhered to the following specifications. The instrument was a Thermo Scientific Corp., USA Trace GC Ultra Gas Chromatograph (Thermo Fisher Scientific, Waltham, MA, USA), to which an ISQ Single Quadrupole Mass Spectrometer (Thermo Fisher Scientific, Waltham, MA, USA) was attached. A TG-WAX MS column (30 m × 0.25 mm i.d., 0.25 μm film thickness) was connected to the GC/MS system. Helium was used as the carrier gas for the analyses, with a split ratio of 1:10 and a temperature program of 40 °C for one minute, a 4.0 °C min^−1^ rise to 160 °C and a hold for six minutes, and a 6.0 °C min^−1^ rise to 210 °C and hold for one minute. At 210 °C, the injector and detector were maintained. In order to inject 1 μL of the mixtures, diluted samples (1:10 hexane, *v*/*v*) were always utilized. By ionizing electrons at 70 eV over a spectral range of *m*/*z* 40–450, the mass spectra were obtained.

#### 2.5.3. Free Proline and Soluble Proteins Content

We used the method in [37] to estimate the free proline content (µmol g^−1^ FW) in fresh moringa leaves, whereby 1 g of fresh leaves was homogenized in aqueous sulfo-salicylic acid (3%). The extract was filtered. We then added a glacial acetic acid and ninhydrin solution at 100 °C for 1 h, followed by terminating the reaction by placing it in an ice bath and mixing it with toluene. The toluene phase was aspirated from the aqueous phase. The contents were determined via the colorimetric method at 520 nm and calculated as µmol g^−1^ fresh weight. Following the instructions in [38], the soluble protein contents (mg g^−1^ FW) of the leaves were measured using Folin–Ciocalteu reagent. Fresh *M. oleifera* leaves (0.5 g) were ground in 1 mL of phosphate-buffered saline with pH 7.2 (10 mM Na_2_HPO_4_, 2 mM KH_2_PO_4_, 2.7 mM KCl, and 1.37 mM NaCl) and 1 µM of cocktail protease inhibitor. After grinding, the plant material was centrifuged at 12,000 rpm for 5 min at room temperature. The supernatant was transferred to another cuvette in order to estimate the total soluble proteins. The absorbance was recorded at 595 nm using a spectrophotometer (UV-4000, O.R.I., Hille, Germany), and the total soluble protein content was quantified by putting absorbance readings into an equation derived from the standard curve.

#### 2.5.4. Total Phenolic Content (mg GAE g^−1^ DW)

First, 1 g of frozen leaves was ground in 50 mL of methanol. The homogenate was shaken for 1 h at room temperature. Then, the extract was filtered. The concentration of total phenols was detected according to Singleton and Rossi [39], using gallic acid as a standard. Next, 1 mL of extract, deionized water (10 mL), and 1 mL of 10% Folin–Ciocalteu phenol were added. Five minutes later, 20% sodium carbonate solution (2.0 mL) was added. Then, the solution was kept in darkness, and the absorbance was measured at 750 nm and expressed as mg gallic acid g^−1^ moringa leaves dry weight.

#### 2.5.5. The Content of Total Flavonoids (mg RE g^−1^ DW)

A 1 g sample of frozen leaves was ground in 50 mL of methanol. The homogenate was shaken for 1 h at room temperature. Then, the extract was filtered. The determination of the total flavonoids was performed by a spectrophotometric method described by Kim et al. [40]. First, 1 mL of the extract was transferred to test tubes wrapped with aluminum foil containing 4 mL distilled water. Subsequently, at time 0, 0.3 mL of 5% sodium nitrite was added to the tubes and vortexed for 10 s. After 5 min, 0.3 mL of 10% aluminum chloride was added, vortexed for 10 s, and left to rest for 6 min. Subsequently, 2 mL of 1 M sodium hydroxide was added to the mixture, and the tubes were vortexed for 10 s. Immediately after, the mixture was diluted by adding 2.4 mL of distilled water and again homogenized in a vortex for 10 s. The concentration of total flavonoids was determined from the optical absorbance values obtained at 510 nm and expressed as mg rutin g^−1^ dry weight.

#### 2.5.6. IC_50_ (µg mL^−1^) Values for Antioxidant Activity

Approximately 1 g of frozen leaves was ground in 50 mL of methanol. The homogenate was shaken for 1 h at room temperature. Then, the extract was filtered. The antiradical activity of different samples was evaluated using the stable 2,2-diphenyl-1-picrylhydrazyl radical (DPPH) [41]. An antioxidant is a chemical compound that can give one or more electrons (electron donor) to a free radical, which can obstruct the free radical reaction. The DPPH test (2,2-diphenyl-1-picrylhydrazyl) uses UV–Vis spectrophotometry due to finding out the antioxidant activity. A compound can be said to have antioxidant activity if the compound can donate its hydrogen atom to bind to DPPH to form a reduced DPPH characterized by looking at the change in color of each sample after incubation with DPPH. This is, increasingly, the loss of purple color. The determination of antioxidant activity is expressed in IC_50_ (μg mL^−1^) as the antioxidant capacity. The IC_50_ value is defined as the efficacy concentration of the test compounds that can reduce free radicals by as much as 50%. The IC_50_ values (μg mL^−1^) for the antioxidant activities were estimated by plotting the extract concentration (x) and DPPH inhibition (y) and fitting the data with a straight line.

### 2.6. Statistical Analysis

SAS v9.1 software was used to perform the ANOVA for the split plot design. The homogeneity of the variances was verified using Hartley’s test. The treatment means were compared using Duncan’s test at *p* = 0.05.

## 3. Results

### 3.1. Oil Parameters

**Figure 1:**
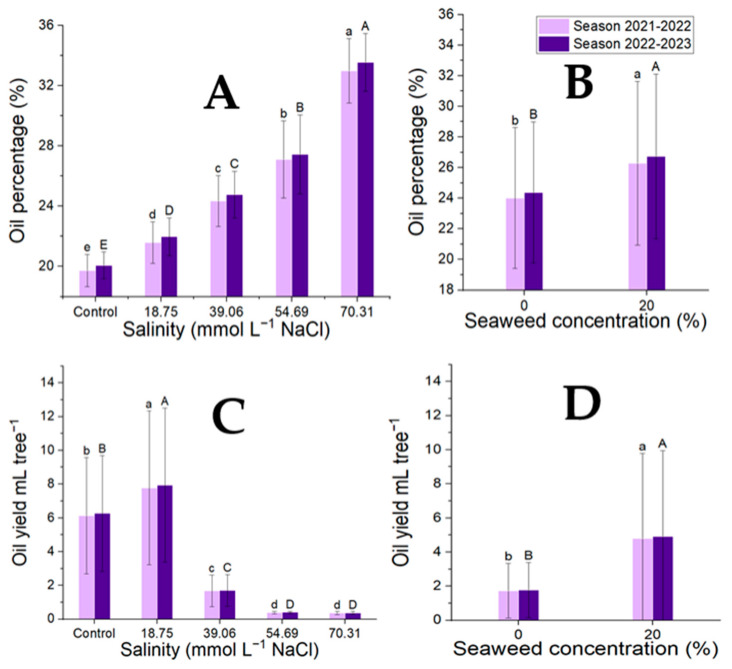
Effect of the salinity levels (**A**,**C**) (regardless of seaweed effect) and seaweed concentrations (**B**,**D**) on the fixed oil content (mL tree^−1^) and fixed oil percentage (%) of *Moringa oleifera* seeds during two seasons. Means with different letters are significantly different at *p* < 0.05.

The impact of the salt levels and seaweed concentrations and their interaction with the oil parameters (the fixed oil yield per tree and fixed oil percentage) were highly significant for all traits. For both characteristics, the greatest concentration of seaweed was 20% (Figure 1). In terms of oil percentage, tap water had the lowest salinity across all seaweed treatments, while the highest level was NaCl 70.31 mmol L^−1^ (Figure 1). The salinity level across the seaweed treatments was the highest at NaCl 18.75 mmol L^−1^ and the lowest at NaCl 70.31 mmol L^−1^ with respect to the fixed oil yield per tree (Figure 1).

The combined treatments of seaweed and salinity are shown in Table 5. The highest oil percentage was found at a concentration of 20% seaweed under a salinity level of NaCl 70.31 mmol L^−1^, and the highest fixed oil yield per tree was found at the same concentration of seaweed under a salinity level of NaCl 18.75 mmol L^−1^, indicating an interaction between the seaweed treatment and salinity levels. The lowest concentration (0%) under tap water had the lowest oil percentage, and the lowest fixed oil yield per tree had the same concentration of seaweed under the salinity level of NaCl 70.31 mmol L^−1^ (Table 5). The correlation between fixed oil percentage and fixed oil yield per tree indicates a significant negative association of these traits (r = −0.61) (*p* < 0.001).

### 3.2. Fixed Oil Analysis of Seeds

**Figure 2:**
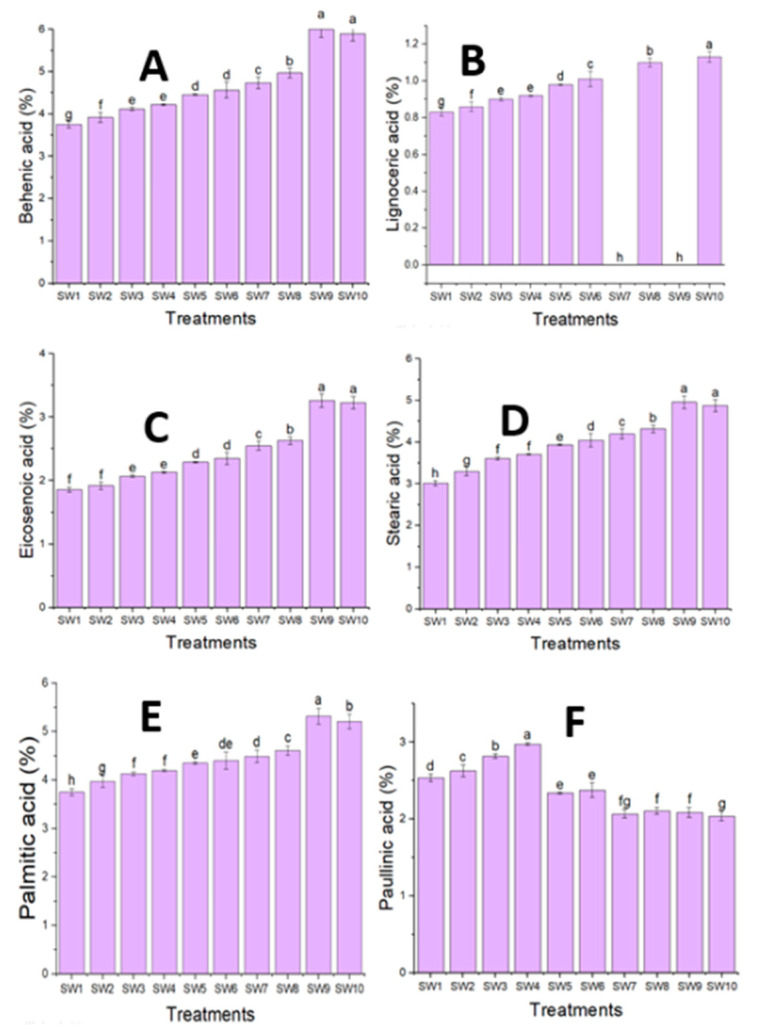
The data are expressed as the average ± SD. Averages with the same letter in the same column are not significant at a 5% probability level, according to Duncan’s test. The behenic (**A**), lignoceric (**B**), eicosenoic (**C**), stearic (**D**), palmitic (**E**), and paullinic (**F**) fatty acid contents in the oil seeds of *Moringa oleifera* under the effects of the salinity levels and seaweed concentrations. The notation of the treatments corresponds to the notation given in Table 3.

**Figure 3:**
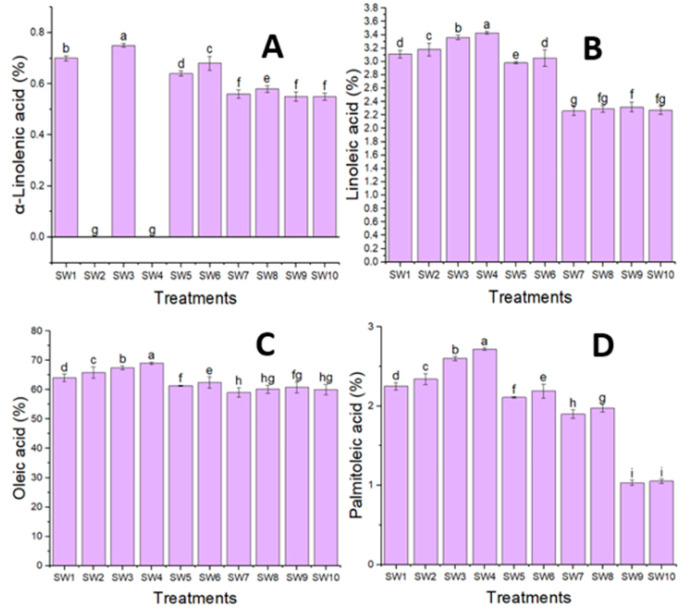
Averages with the same letter in the same column for each season are not significant at a 5% probability level, according to Duncan’s test. The α-linolenic acid (**A**), linoleic acid (**B**), oleic acid (**C**), and palmitoleic (**D**) fatty acid contents in the oil seeds of *Moringa oleifera* under the effects of the salinity levels and seaweed concentrations. The notation of the treatments corresponds to the notation given in Table 3.

Paullinic acid, α-linolenic acid, oleic acid, and palmitoleic acid comprise the majority of unsaturated fatty acids in the fixed oil of *M. oleifera*, with a combined percentage ranging from 65.87 to 78.19%, according to the data shown in Figure 2 and Figure 3. However, lignoceric acid, behenic acid, eicosenoic acid, palmitic acid, and stearic acid—which ranged from 13.23 to 20.34%—were the primary saturated fatty acids found in the seeds of this plant. The fatty acid content was investigated using GC/MS, and the percentage of total identified fatty acids ranged from 81.86 to 93.37%. Additionally, it was discovered that the highest percentages of unsaturated fatty acids in *M. oleifera* fixed oil were present at a salinity level of 18.75 mmol L^−1^ NaCl. Furthermore, the highest percentages of oleic acid (69.06%), linoleic acid (3.43%), palmitoleic acid (2.72%), and phthalic acid (2.98%) were observed in the treatment comprising 18.75 mmol L^−1^ NaCl salinity plus 20% seaweed. A combination of 0% seaweed and 18.75 mmol L^−1^ NaCl salinity produced the highest α-linolenic acid percentage (0.76%). The obtained data unequivocally demonstrated that, as the salinity levels rose, so did the percentage of saturated fatty acids. The percentage of unsaturated fatty acids peaked when the salinity level was NaCl 18.75 mmol L^−1^, but it declined as the salinity level rose.

### 3.3. Protein and Proline Contents

**Figure 4:**
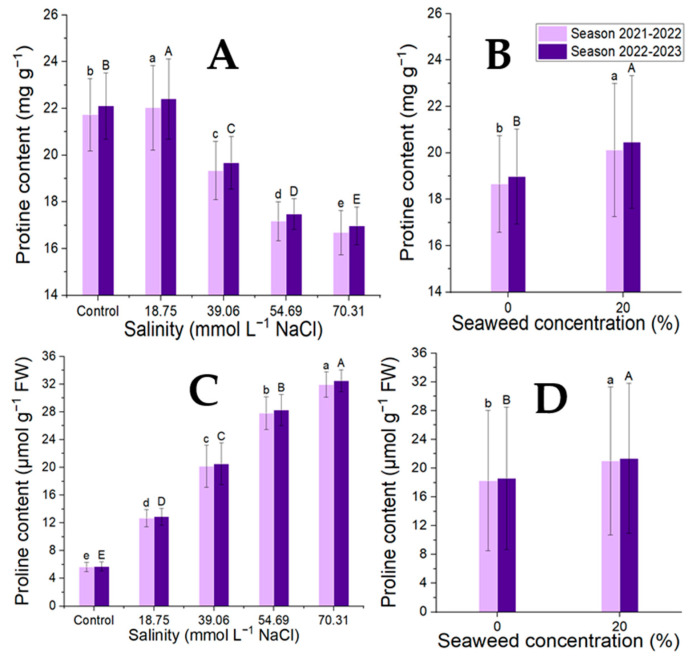
Effect of the salinity levels (**A**,**C**) (regardless of seaweed effect) and seaweed concentrations (**B**,**D**) (regardless of salinity levels) on the proline and protein contents of *M. oleifera* leaves in the two seasons of the study. Means with different letters are significantly different at *p* < 0.05.

The findings in Figure 4 illustrate how applications of seaweed extract and salinity affected the proline and protein contents in terms of the osmoprotectant concentrations. The levels of salinity and protein showed a significant negative correlation. The proline content and salinity levels showed a positive and significant correlation, with the proline levels significantly increasing as the salinity levels increased. Applying seaweed extract topically to moringa leaves greatly raised their concentrations of proline and protein. In terms of the interaction effect, moringa plants grown in the first and second seasons under NaCl 18.75 mmol L^−1^ salinity had the highest protein content (23.37 and 23.77 mg g^−1^); however, as the salinity level rose, this value progressively dropped, reaching 17.10 and 17.40 mg g^−1^ under the SW7 treatment. On the other hand, the moringa leaves grown in higher salinity levels and treated with seaweed extract exhibited the highest proline values (32.84 and 33.40 µmol g^−1^ FW) in the first and second seasons, respectively (Table 6).

### 3.4. Bioactive Compound: Total Phenolic and Flavonoids Contents and Antioxidant Activity

**Figure 5:**
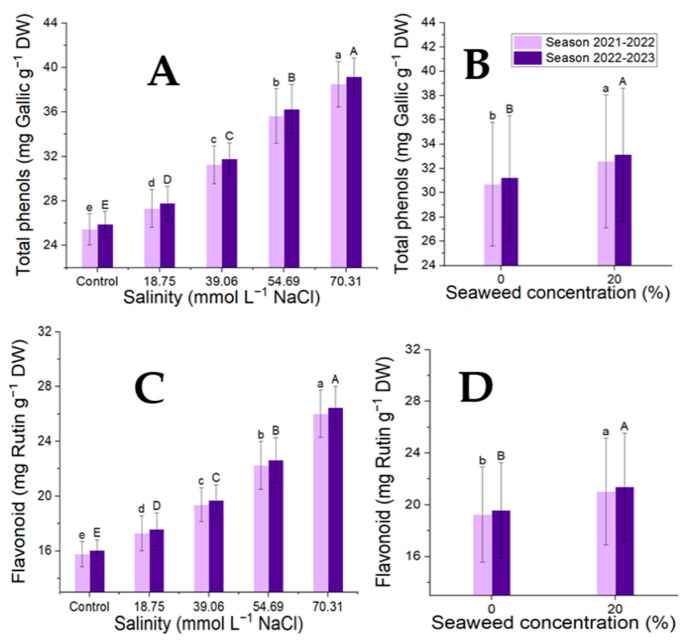
The effect of the salinity levels (**A**,**C**) (regardless of seaweed effect) and seaweed concentrations (**B**,**D**) (regardless of salinity levels) on the total phenols and flavonoids contents of *M. oleifera* leaves in the two seasons of the study. Means with different letters are significantly different at *p* < 0.05.

**Figure 6:**
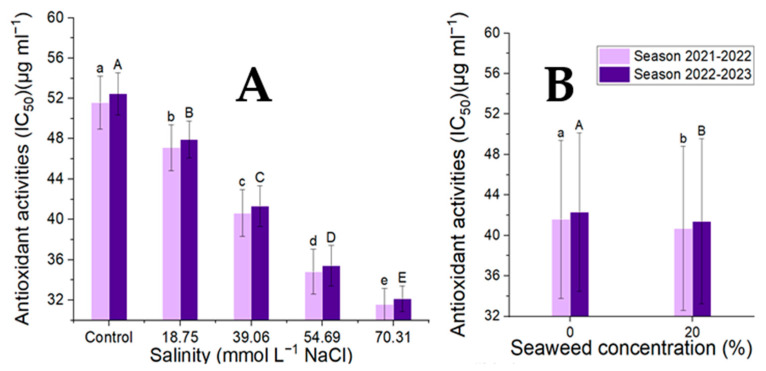
Effect of the salinity levels (**A**) (regardless of seaweed effect) and seaweed concentrations (**B**) (regardless of salinity levels) on the antioxidant activities (IC_50_) of *M. oleifera* leaves during the study. Means with different letters are significantly different at *p* < 0.05.

The seaweed concentrations, salinity levels, and their combinations all significantly affected the antioxidant activity and secondary products of the moringa tree characteristics. The highest concentration of seaweed for each of these features was found at 20% (Figure 5 and Figure 6; Table 7 and Table 8). Among the seaweed treatments, tap water had the highest levels of total phenolics, flavonoids, and antioxidant activity and the lowest salinity (70.31 mmol L^−1^ NaCl). At a salinity level of 70.31 mmol L^−1^ NaCl, seaweed (20%) exhibited the highest concentration of flavonoids, total phenolic content, and antioxidant activity, while seaweed (0%) with tap water had the lowest concentration of these properties. The association between the total phenolic contents, flavonoid contents, and antioxidant activity was extremely significant (*p* < 0.001). The strongest correlation was between the total phenolic and flavonoid contents, total phenolic contents, and antioxidant activities, as well as flavonoid contents and antioxidant activities (r = 0.98, 0.89, and 0.88).

## 4. Discussion

### 4.1. Effects of Salinity

Salinity stress, which jeopardizes plant growth and productivity, is one of the primary factors limiting crop productivity worldwide. The current study found that the maximum oil yield per tree was produced by the moringa trees grown at the salinity level of NaCl 18.75 mmol L^−1^. Moringa trees grow to a height of 10 to 12 m very quickly, and they can be either deciduous or evergreen [42]. Due to the dilution effect, which lessened the harmful effects of elevated Na^+^ ions, plants with rapid growth demonstrated some tolerance to salinity [43]. Fast growth lessens the chance of salt stress and encourages steady growth, which increases yield and biomass. As such, the growth response functions as a useful marker for assessing the tolerance of plants to stress [44]. According to [45], a pepper plant’s growth increased steadily as the salinity levels rose because of the cells’ adaptive osmotic pressure regulation reactions to high salinity. However, because of the high salinity, the low productivity of the moringa trees meant that less oil was produced per tree. According to [46], salinity inhibits cell division and growth, as well as physiological and metabolic processes. To regulate the osmotic potential, plants accumulated proteins and other compatible solutes, such as proline. Stress-induced proline accumulation maintains the integrity of the cell by adjusting the osmotic force of the cytosol to that of the vacuole and the surrounding environment. According to [47], proline is important for providing osmoprotection, stabilizing enzyme structures, and defending against reactive oxygen species. Prior studies [48,49,50] discovered that tobacco, canola, and coriander plants had higher proline contents when exposed to saline conditions. These results are in line with those of the moringa plants, which exhibited an increase in proline content as the NaCl stress increased. The amount of soluble protein was higher at the lowest salinity level and, subsequently, decreased as the salinity increased. The authors of [51] observed that, at 50- and 100-mM salt concentrations, there was a comparable rise in the proline content, along with a decrease in soluble protein. This study found that, as the salinity increased, so did the levels of flavonoids, polyphenols, and antioxidant activity. The osmotic effect caused by salt depletes the available water and frequently induces oxidative stress in cells, which releases free oxygen radicals to channel excess delay and restrict plant growth and development differentiation. This is accomplished by reducing the power produced as a result of a decline in the pathways that control photosynthesis, respiration, and mitochondrial respiration or the photosynthetic dark reaction [52,53]. Several studies on vascular plants, including legumes, have shown that the detrimental effects of salt-induced oxidative stress are caused by high ROS production [54,55].

### 4.2. Effects of Seaweed Extract

The oil yield of *M. oleifera* trees was enhanced by applying *Ulva lactuca* seaweed extract in comparison to the control treatment. It may be due to the high concentrations of microelements, organic matter, fatty acids, and vitamins, as auxins, gibberellins, and cytokinin are among the growth regulators that are abundant in seaweed extract [56]. These findings are consistent with Gharib et al. [57], who discovered that, when rosemary plants were sprayed with 20% and 40% seaweed extract, the vegetative parameters improved and the oil percentage increased in two cuttings, respectively. Amer and Atteya [24] reported that the application of seaweed extract significantly raised the percentage of seed oil and the yield per plant of *Hibiscus sabdariffa* plants as compared to the control treatment.

Salt is known to inhibit plant growth, and saline soils and irrigation pose serious obstacles to crop productivity [58]. As salinity rises, salt stress can restrict and impede plant growth and development differentiation [59]. Due to their nontoxic nature and environmental friendliness, seaweed extracts are becoming more and more popular as low-cost substitutes for conventional fertilizers [54,60,61]. This study’s objective was to evaluate how well *U. lactuca* seaweed extract could support *M. oleifera* tree productivity in terms of oil yield and secondary products when the trees were under salinity stress. Seaweed extracts belonging to different species have been seen to have a mitigating impact on plants subjected to salt stress. This is attributed to the cumulative action of various chemicals, including polyamines, phenolics, protein, carbohydrates, and betaines. The goal of these substances is to encourage the synthesis and accumulation of ameliorative molecules [62]. The study’s findings show that the seaweed *U. lactuca* was able to considerably increase the oil yield and secondary products of *M. oleifera* trees grown in salinity stress and control environments. Salinity stress has a negative impact on plant growth and seed yield [63], which reduces the seed oil yield of *M. oleifera* trees, because it directly inhibits cell division and expansion [64]. It was found that applying the seaweed extract reduced the NaCl-induced inhibition of plant growth. Under both control and salinity stress circumstances, it was discovered that applying seaweed derived from *U. lactuca* increased the oil yield [65]. This could have been caused by the presence of proline, total carbohydrates, phenolic chemicals, and growth-promoting agents. Our results also corroborate those of a study by the authors of [66], which found that applying *U. lactuca* seaweed extract under salinity stress conditions increased the sunflower plant’s seed oil yield. In the current study, seaweed applications and salinity stress had an effect on all fatty acids. Elevating the salinity levels had an impact on the high-oleate cultivars’ fatty acid composition, which decreased the concentration of oleic acid [67]. The accumulation of active oxygen species in salinized plant cells leads to lipid peroxidation, oxidative stress, and distortion of the cellular redox system [68]. Lipid peroxidation, which modifies the permeability and fluidity of membranes, causes lipid bilayer malfunction. Plant membranes are rich in linoleic and linolenic acids, which are essential lipoxygenase substrates [69]. In response to salinity stress, polyunsaturated fatty acids decrease, and saturated fatty acids increase. Because plants can reorganize their cell membranes to contain fewer polyunsaturated fatty acids in order to protect their cells from the harmful oxidative effects of salt ions, it is possible that drops in desaturase activity, which occur as an adaptive response to salt stress, are the cause of increases in saturated fatty acid [70,71]. Membrane fluidity [72,73] and Na and Cl ion permeability [74] were both hindered by low unsaturation degrees. A significant concentration of total nonstructural carbohydrates, which are associated with increased proline and photochemical efficiency, was found when the authors of [75] investigated the effects of salt stress on *Paspalum vaginatum* caused by *Ascophyllum nodosum* seaweed. In response to abiotic stress, plants generate secondary metabolites and antioxidants that reduce the damage they sustain [76]. Since oxidative damage is the primary cause of salinity stress damage, seaweed has been used extensively to mitigate this damage. In this study, the application of seaweed extract under salinity stress increased *M. oleifera* trees’ antioxidant activity and their secondary products (flavonoids and polyphenols) and osmoprotectants (proline and protein). This can be linked to the improved biochemical characteristics that resulted from seaweed extract application, which increased the synthesis of essential plant biochemicals such as protein, phenol, and antioxidant contents. *A. nodosum* seaweed extracts were used in a study [75] to determine the increased proline accumulation, antioxidant capacity, and phenolic and flavonoid contents in *Spiraea nipponica* and *Pittosporum eugenioides*. The growth-stimulating activities and the antioxidant-rich macro- and microelements, vitamins, auxins, and phytohormones found in seaweed were ascribed to these results. The seaweed extract’s bioactive components, which include phenols, carbohydrates, protein, and proline, have a positive effect on plant growth by enhancing the production of metabolites (amino acids that reduce stress), the membrane permeability, and the movement of osmolytes and ions. These processes improve plants’ resistance to abiotic stress by altering their water capacity and turgor pressure [30]. Proline is necessary for osmoregulation and has been shown to have antioxidant properties. The synthesis of ROS and signaling pathways are significantly influenced by proline metabolism [77]. Proline has been proposed as a flag molecule that responds to salt; when plants are stressed by salinity, this amino acid is the most prevalent, and its concentration rises significantly [78]. The plants under salinity stress exhibited an elevated proline content, in line with previous studies. Research has demonstrated that different plant species have different concentrations of osmolytes and antioxidant activity; tomatoes [79], potatoes [80], and melons [81] are examples of this phenomenon. Furthermore, these authors reported that proline accumulated in salt-tolerant cultivars and suggested that decreased proline dehydrogenase activity and an increased activity of proline synthesis enzymes were the causes of this phenomenon rather than protein degradation [82]. Furthermore, according to these authors, proline does not raise the salinity tolerance, because it is a stress indicator. According to [13], proline application effectively increased plants’ ability to withstand stress. In this study, we found that, when salinity stress was applied to moringa plants, the concentration of proline increased. However, this increase was greater when the seaweed extract was applied to moringa plants, which may be because the seaweed extract contains amino acids, as well as proline as one of its amino acids. 

## 5. Conclusions

These findings suggest that seaweed extract can help *Moringa oleifera* trees overcome salt stress by promoting plant growth. The levels of flavonoids, polyphenols, osmoprotectants, and antioxidant activity were all markedly increased by the seaweed extract. As a result of applying seaweed extract, there was an increase in the yield of moringa seed oil and an improvement in the plant’s resistance to salinity stress.

Based on these results, we recommend conducting more studies on the effect of salinity and seaweed extracts on the components of the *Moringa oleifera* tree.

## Figures and Tables

**Table 1:** Physical and chemical properties of the soil under experimentation.

Characteristic							
O.M.	1.25	Clay	27%	Soluble ions			
		Sand	37%				
pH	7.9	Silt	36%	HCO_3_^−^	2.33 mg kg^−1^	Ca^2+^	6.00 mg kg^−1^
EC *	2.24 mS cm^−1^	Soil typs	Loam	K^+^	0.30 mg kg^−1^	Na^+^	4.38 mg kg^−1^

* Electrical conductivity.

**Table 2:** The chemical composition of the irrigation water under investigation.

Parameters	pH	EC	Ca^2+^	Mg^2+^	Na^+^	K^+^	HCO^3−^	Cl^−^	SO_4_^−2^
Values	7.6	0.45 dS m^−1^	0.56 mmol L^−1^	0.27 mmol L^−1^	2.53 mmol L^−1^	0.22 mmol L^−1^	0.04 mmol L^−1^	2.87 mmol L^−1^	0.62 mmol L^−1^

**Table 3:** The following were all the various combinations of seaweed treatments and salinity.

Treatments	Number of Repetitions Treatment ^−1^	Salinity		Seaweed (%)
		EC (dS m^−1^)	(mmol L^−1^ NaCl)	
SW1	N = 9	Tap water	Tap water	0
SW2	N = 9			20
SW3	N = 9	1.71	18.75	0
SW4	N = 9			20
SW5	N = 9	3.57	39.06	0
SW6	N = 9			20
SW7	N = 9	4.99	54.69	0
SW8	N = 9			20
SW9	N = 9	6.42	70.31	0
SW10	N = 9			20

**Table 4:** Chemical properties of the extracts of dried seaweed from *Ulva lactuca*.

Parameters	Amino Acids	Lipid	Zn	K	Mg	Ca	Fe
Values	8.9%	5.6%	3 ppm	13 ppm	46 ppm	43 ppm	2 ppm

**Table 5:** Fixed oil percentage (%) and fixed oil content (ml plant^−1^) of *M. oleifera* seeds as impacted by salinity and seaweed combination treatments during the study.

Salinity (mmol L^−1^ NaCl) and Seaweed (%) Combination Treatments	Fixed Oil Percentage (%)		Fixed Oil Content (ml Plant^−1^)	
	Season 2021–2022	Season 2022–2023	Season 2021–2022	Season 2022–2023
SW1 *	19.37 ± 23.77 g	19.70 ± 0.91 i	3.20 ± 1.36 c	3.27 ± 1.13 c
SW2	20.09 ± 23.12 fg	20.44 ± 0.85 h	9.06 ± 3.77 b	9.25 ± 3.16 b
SW3	20.74 ± 21.09 f	21.10 ± 0.86 g	3.88 ± 1.59 c	3.96 ± 1.29 c
SW4	22.44 ± 21.03 e	22.83 ± 0.95 f	11.67 ± 4.86 a	11.92 ± 4.07 a
SW5	23.21 ± 20.46 e	23.61 ± 0.96 e	0.87 ± 0.36 de	0.89 ± 0.29 d
SW6	25.48 ± 18.88 d	25.91 ± 1.08 d	2.47 ± 1.03 cd	2.52 ± 0.86 c
SW7	25.10 ± 17.54 d	25.28 ± 1.28 d	0.38 ± 0.16 e	0.39 ± 0.14 d
SW8	29.10 ± 17.45 c	29.60 ± 1.23 c	0.40 ± 0.17 e	0.40 ± 0.14 d
SW9	31.66 ± 17.40 b	32.21 ± 1.28 b	0.31 ± 0.13 e	0.32 ± 0.11 d
SW10	34.30 ± 16.49 a	34.88 ± 1.46 a	0.41 ± 0.17 e	0.41 ± 0.14 d

The data are expressed as the average ± SD. Averages with the same letter in the same column for each season are not significant at a 5% probability level, according to Duncan’s test. * The notation of the treatments in this table corresponds to the notation given in Table 3.

**Table 6:** Protein and proline contents of *M. oleifera* leaves as affected by the salinity and seaweed combination treatments during the study’s two seasons.

Salinity (mmol L^−1^ NaCl) and Seaweed (%) Combination Treatments	Protein Content (mg g^−1^ FW)		Proline Content (µmol g^−1^ FW)	
	Season 2021–2022	Season 2022–2023	Season 2021–2022	Season 2022–2023
SW1 *	20.73 ± 1.20 c	21.09 ± 0.97 c	5.08 ± 0.29 i	5.17 ± 0.24 j
SW2	22.73 ± 1.23 b	23.12 ± 0.97 b	6.19 ± 0.34 i	6.30 ± 0.27 i
SW3	20.68 ± 1.07 c	21.03 ± 0.85 c	11.70 ± 0.61 h	11.90 ± 0.48 h
SW4	23.37 ± 1.26 a	23.77 ± 0.99 a	13.67 ± 0.74 g	13.90 ± 0.58 g
SW5	18.56 ± 0.96 e	18.88 ± 0.77 e	17.55 ± 0.90 f	17.85 ± 0.73 f
SW6	20.12 ± 1.08 d	20.46 ± 0.86 d	22.77 ± 1.23 e	23.16 ± 0.97 e
SW7	17.10 ± 0.92 f	17.40 ± 0.73 f	26.02 ± 1.40 d	26.46 ± 1.11 d
SW8	17.25 ± 0.93 f	17.54 ± 0.73 f	29.60 ± 1.60 c	30.11 ± 1.26 c
SW9	16.21 ± 0.85 g	16.49 ± 0.66 g	31.04 ± 1.62 b	31.57 ± 1.26 b
SW10	17.15 ± 0.93 f	17.45 ± 0.73 f	32.84 ± 1.77 a	33.40 ± 1.40 a

The data are expressed as the average ± SD. Averages with the same letter in the same column for each season are not significant at a 5% probability level, according to Duncan’s test. * The notation of the treatments in this table corresponds to the notation given in Table 3.

**Table 7:** The salinity and seaweed combination treatments’ mean values for the total phenol and flavonoid contents of *M. oleifera* leaves during the study seasons.

Salinity (mmol L^−1^ NaCl) and Seaweed (%) Combination Treatments	Total Phenols (mg GAE g^−1^ DW)		Flavonoids (mg RE g^−1^ DW)	
	Season 2021–2022	Season 2022–2023	Season 2021–2022	Season 2022–2023
SW1 *	24.93 ± 1.45 h	25.35 ± 2.34 h	15.34 ± 0.89 h	15.60 ± 0.72 h
SW2	25.97 ± 1.40 g	26.42 ± 2.21 g	16.19 ± 0.87 g	16.47 ± 0.69 g
SW3	26.33 ± 1.36 g	26.78 ± 2.17 g	16.38 ± 0.84 g	16.66 ± 0.68 g
SW4	28.32 ± 1.53 f	28.81 ± 2.40 f	18.19 ± 0.98 f	18.50 ± 0.78 f
SW5	30.50 ± 1.57 e	31.02 ± 2.52 e	18.61 ± 0.96 f	18.93 ± 0.77 f
SW6	32.01 ± 1.73 d	32.55 ± 2.72 d	20.13 ± 1.08 e	20.47 ± 0.86 e
SW7	34.06 ± 1.84 c	34.65 ± 2.89 c	20.98 ± 1.14 d	21.34 ± 0.89 d
SW8	37.23 ± 2.01 b	37.86 ± 3.16 b	23.50 ± 1.27 c	23.90 ± 1.00 c
SW9	37.68 ± 1.96 b	38.32 ± 3.05 b	24.93 ± 1.30 b	25.35 ± 1.01 b
SW10	39.35 ± 2.12 a	40.01 ± 3.34 a	27.10 ± 1.46 a	27.56 ± 1.15 a

The data are expressed as the average ± SD. Averages with the same letter in the same column for each season are not significant at a 5% probability level, according to Duncan’s test. * The notation of the treatments in this table corresponds to the notation given in Table 3.

**Table 8:** The average values of the antioxidant activity (IC_50_) of *Moringa oleifera* leaves under the combined treatments of seaweed and salinity during the two study seasons.

Salinity (mmol L^−1^ NaCl) and Seaweed (%) Combination Treatments	Antioxidant Activities (IC_50_) * (µg mL^−1^)	
	Season 2021–2022	Season 2022–2023
SW1 *	51.25 ± 2.98 a	52.12 ± 2.40 a
SW2	51.92 ± 2.80 a	52.80 ± 2.21 a
SW3	47.55 ± 2.45 b	48.37 ± 1.97 b
SW4	46.67 ± 2.52 b	47.46 ± 1.98 b
SW5	41.82 ± 2.15 c	42.53 ± 1.73 c
SW6	39.46 ± 2.13 d	40.13 ± 1.67 d
SW7	36.16 ± 1.95 e	36.77 ± 1.54 e
SW8	33.47 ±1.80 f	34.03 ± 1.42 f
SW9	31.18 ± 1.63 g	31.71 ± 1.26 g
SW10	32.00 ± 1.73 g	32.54 ± 1.36 g

The data are expressed as the average ± SD. Averages with the same letter in the same column for each season are not significant at a 5% probability level, according to Duncan’s test. * The concentration (µg/mL) for 50% inhibition. * The notation of the treatments in this table corresponds to the notation given in Table 3.

## Data Availability

The data presented in this study are available within the article.

## References

[1] Ramachandran C., Peter K.V., Gopalakrishnan P.K. (1980). Drumstick (*Moringa oleifera*): A Multipurpose Indian Vegetable. Econ. Bot..

[2] Morton J.F. (1991). The Horseradish Tree, Moringa Pterygosperma (Moringaceae)—A Boon to Arid Lands?. Econ. Bot..

[3] Rockwood J.L., Anderson B.G., Casamatta D.A. (2013). Potential Uses of *Moringa oleifera* and an Examination of Antibiotic Efficacy Conferred by *M. oleifera* Seed and Leaf Extracts Using Crude Extraction Techniques Available to Underserved Indigenous Populations. Int. J. Phytother. Res..

[4] Warra A.A. (2014). A Review of *Moringa oleifera* Lam. Seed Oil Prospects in Personal Care Formulations. Res. Rev. J. Pharm. Nanotechnol.

[5] Nadeem M., Imran M. (2016). Promising Features of *Moringa oleifera* Oil: Recent Updates and Perspectives. Lipids Health Dis..

[6] Aiyelaagbe I.O.O. Nigerian Horticulture: Facing the Challenges of Human Health and Agricultural Productivity. Proceedings of the Keynote Address Presented at the 29th Annual National Conference of Horticultural Society of Nigeria.

[7] Fuglie L.J. (1999). The Miracle Tree: Moringa oleifera, Natural Nutrition for the Tropics.

[8] Singh J., Thakur J.K. (2018). Photosynthesis and Abiotic Stress in Plants. Biotic and Abiotic Stress Tolerance in Plants.

[9] Flowers T.J., Gaur P.M., Gowda C.L.L., Krishnamurthy L., Samineni S., Siddique K.H.M., Turner N.C., Vadez V., Varshney R.K., Colmer T.D. (2010). Salt Sensitivity in Chickpea. Plant Cell Environ..

[10] Youssef A.M. (2009). Salt Tolerance Mechanisms in Some Halophytes from Saudi Arabia and Egypt. Res. J. Agric. Biol. Sci..

[11] Dos Santos T.B., Ribas A.F., de Souza S.G.H., Budzinski I.G.F., Domingues D.S. (2022). Physiological Responses to Drought, Salinity, and Heat Stress in Plants: A Review. Stresses.

[12] Liang W., Ma X., Wan P., Liu L. (2018). Plant Salt-Tolerance Mechanism: A Review. Biochem. Biophys. Res. Commun..

[13] Bacha H., Tekaya M., Drine S., Guasmi F., Touil L., Enneb H., Triki T., Cheour F., Ferchichi A. (2017). Impact of Salt Stress on Morpho-Physiological and Biochemical Parameters of *Solanum lycopersicum* Cv. Microtom leaves. S. Afr. J. Bot..

[14] Munné-Bosch S. (2005). The Role of α-Tocopherol in Plant Stress Tolerance. J. Plant Physiol..

[15] Rai G.K., Rai N.P., Rathaur S., Kumar S., Singh M. (2013). Expression of Rd29A: AtDREB1A/CBF3 in Tomato Alleviates Drought-Induced Oxidative Stress by Regulating Key Enzymatic and Non-Enzymatic Antioxidants. Plant Physiol. Biochem..

[16] Talbi S., Romero-Puertas M.C., Hernández A., Terrón L., Ferchichi A., Sandalio L.M. (2015). Drought Tolerance in a Saharian Plant Oudneya Africana: Role of Antioxidant Defences. Environ. Exp. Bot..

[17] Puniran-Hartley N., Hartley J., Shabala L., Shabala S. (2014). Salinity-Induced Accumulation of Organic Osmolytes in Barley and Wheat Leaves Correlates with Increased Oxidative Stress Tolerance: In Planta Evidence for Cross-Tolerance. Plant Physiol. Biochem..

[18] De Vasconcelos A.C.F., Chaves L.H.G. (2019). Biostimulants and Their Role in Improving Plant Growth under Abiotic Stresses. Biostimulants in Plant Science.

[19] Ali O., Ramsubhag A., Jayaraman J. (2021). Biostimulant Properties of Seaweed Extracts in Plants: Implications towards Sustainable Crop Production. Plants.

[20] Mukherjee A., Patel J.S. (2020). Seaweed Extract: Biostimulator of Plant Defense and Plant Productivity. Int. J. Environ. Sci. Technol..

[21] Drira M., Mohamed J.B., Hlima H.B., Hentati F., Michaud P., Abdelkafi S., Fendri I. (2021). Improvement of Arabidopsis Thaliana Salt Tolerance Using a Polysaccharidic Extract from the Brown Algae Padina Pavonica. Algal Res..

[22] Bayomy H.M., Alamri E.S. (2024). Biochemical Assessments of Six Species of Edible Coastal Algae Collected from Tabuk Region in Saudi Arabia. Molecules.

[23] Zou P., Lu X., Zhao H., Yuan Y., Meng L., Zhang C., Li Y. (2019). Polysaccharides Derived from the Brown Algae Lessonia Nigrescens Enhance Salt Stress Tolerance to Wheat Seedlings by Enhancing the Antioxidant System and Modulating Intracellular Ion Concentration. Front. Plant Sci..

[24] Atteya A.K.G., Amer H.M. (2018). Influence of Seaweed Extract and Amino Acids on Growth, Productivity and Chemical Constituents of *Hibiscus sabdariffa* L. Plants. Biosci. Res..

[25] Layek J., Das A., Idapuganti R.G., Sarkar D., Ghosh A., Zodape S.T., Lal R., Yadav G.S., Panwar A.S., Ngachan S. (2018). Seaweed Extract as Organic Bio-Stimulant Improves Productivity and Quality of Rice in Eastern Himalayas. J. Appl. Phycol..

[26] Sharma L., Banerjee M., Malik G.C., Gopalakrishnan V.A.K., Zodape S.T., Ghosh A. (2017). Sustainable Agro-Technology for Enhancement of Rice Production in the Red and Lateritic Soils Using Seaweed Based Biostimulants. J. Clean. Prod..

[27] Franzoni G., Cocetta G., Prinsi B., Ferrante A., Espen L. (2022). Biostimulants on Crops: Their Impact under Abiotic Stress Conditions. Horticulturae.

[28] Carillo P., Ciarmiello L.F., Woodrow P., Corrado G., Chiaiese P., Rouphael Y. (2020). Enhancing Sustainability by Improving Plant Salt Tolerance through Macro-and Micro-Algal Biostimulants. Biology.

[29] Bose J., Rodrigo-Moreno A., Shabala S. (2014). ROS Homeostasis in Halophytes in the Context of Salinity Stress Tolerance. J. Exp. Bot..

[30] Khan Z., Gul H., Rauf M., Arif M., Hamayun M., Ud-Din A., Sajid Z.A., Khilji S.A., Rehman A., Tabassum A. (2022). Sargassum Wightii Aqueous Extract Improved Salt Stress Tolerance in Abelmoschus Esculentus by Mediating Metabolic and Ionic Rebalance. Front. Mar. Sci..

[31] Cottenie A., Verloo M., Kiekens L., Velghe G., Camerlynck R. (1982). Chemical Analysis of Plants and Soils.

[32] Jackson M.L. (1973). Soil Chemical Analysis.

[33] Yasmeen A., Basra S., Ahmed M., Wahid A., Nouman W., Rehman H.U.R. (2013). Exploring the Potential of *Moringa oleifera* Leaf Extract (MLE) as a Seed Priming Agent in Improving Wheat Performance. Turk. J. Bot..

[34] AOAC (2005). AOAC Official Methods of Analysis.

[35] Bharathi S., Dinesh Kumar S., Sekar S., Santhanam P., Divya M., Krishnaveni N., Pragnya M., Dhanalakshmi B. (2021). Experimental Evaluation of Seaweeds Liquid Extracts as an Alternative Culture Medium on the Growth and Proximate Composition of Picochlorum Maculatum. Proc. Natl. Acad. Sci. India Sect. B-Biol. Sci..

[36] Horwitz W. (1975). Official Methods of Analysis.

[37] Bates L.S., Waldren R.P.A., Teare I.D. (1973). Rapid Determination of Free Proline for Water-Stress Studies. Plant Soil.

[38] Bradford M.M. (1976). A Rapid and Sensitive Method for the Quantitation of Microgram Quantities of Protein Utilizing the Principle of Protein-Dye Binding. Anal. Biochem..

[39] Singleton V.L., Rossi J.A. (1965). Colorimetry of Total Phenolics with Phosphomolybdic-Phosphotungstic Acid Reagents. Am. J. Enol. Vitic..

[40] Kim D.-O., Chun O.K., Kim Y.J., Moon H.-Y., Lee C.Y. (2003). Quantification of Polyphenolics and Their Antioxidant Capacity in Fresh Plums. J. Agric. Food Chem..

[41] Brand-Williams W., Cuvelier M.E., Berset C. (1995). Use of a Free Radical Method to Evaluate Antioxidant Activity. LWT-Food Sci. Technol..

[42] Atteya A.K.G., Albalawi A.N., Bayomy H.M., Alamri E.S., Genaidy E.A.E. (2022). Maximizing Leaves, Inflorescences, and Chemical Composition Production of *Moringa Oleifera* Trees under Calcareous Soil Conditions. Plants.

[43] Yeo A.R., Yeo M.E., Flowers S.A., Flowers T.J. (1990). Screening of Rice (Oryza sativa L.) Genotypes for Physiological Characters Contributing to Salinity Resistance, and Their Relationship to Overall Performance.

[44] Akter M., Oue H. (2018). Effect of Saline Irrigation on Accumulation of Na^+^, K^+^, Ca^2+^, and Mg^2+^ Ions in Rice Plants. Agriculture.

[45] Al-Hattab Z.N., Al-Ajeel S.A., El-Kaaby E.A. (2015). Effect of Salinity Stress on Capsicum Annuum Callus Growth, Regeneration and Callus Content of Capsaicin, Phenylalanine, Proline and Ascorbic Acid. J. Life Sci..

[46] Debez A., Chaibi W., Bouzid S. (2001). Effect of NaCl and Growth Regulators on Germination of *Atriplex halimus* L.. Cah. Agric..

[47] Alzahrani S.M., Alaraidh I.A., Migdadi H., Alghamdi S., Khan M.A., Ahmad P. (2019). Physiological, Biochemical, and Antioxidant Properties of Two Genotypes of Vicia Faba Grown under Salinity Stress. Pak. J. Bot..

[48] ÇELİK Ö., Atak C. (2012). The Effect of Salt Stress on Antioxidative Enzymes and Proline Content of Two Turkish Tobacco Varieties. Turk. J. Biol..

[49] Meriem B.F., Kaouther Z., Chérif H., Tijani M., André B. (2014). Effect of Priming on Growth, Biochemical Parameters and Mineral Composition of Different Cultivars of Coriander (*Coriandrum sativum* L.) under Salt Stress. J. Stress Physiol. Biochem..

[50] Sharif P., Seyedsalehi M., Paladino O., Van Damme P., Sillanpää M., Sharifi A.A. (2018). Effect of Drought and Salinity Stresses on Morphological and Physiological Characteristics of Canola. Int. J. Environ. Sci. Technol..

[51] Menezes R.V., de Azevedo A.D., de Oliveira Ribeiro M., Cova A.M.W. (2017). Growth and Contents of Organic and Inorganic Solutes in Amaranth under Salt Stress. Pesqui. Agropecu. Trop..

[52] Abogadallah G.M., Serag M.M., Quick W.P. (2010). Fine and Coarse Regulation of Reactive Oxygen Species in the Salt Tolerant Mutants of Barnyard Grass and Their Wild-type Parents under Salt Stress. Physiol. Plant.

[53] Hsu S.-Y., Kao C.H. (2003). Differential Effect of Sorbitol and Polyethylene Glycol on Antioxidant Enzymes in Rice Leaves. Plant Growth Regul..

[54] Hernández-Herrera R.M., Santacruz-Ruvalcaba F., Ruiz-López M.A., Norrie J., Hernández-Carmona G. (2014). Effect of Liquid Seaweed Extracts on Growth of Tomato Seedlings (*Solanum lycopersicum* L.). J. Appl. Phycol..

[55] Mittova V., Guy M., Tal M., Volokita M. (2004). Salinity Up-regulates the Antioxidative System in Root Mitochondria and Peroxisomes of the Wild Salt-tolerant Tomato Species *Lycopersicon pennellii*. J. Exp. Bot..

[56] Crouch I.J., Van Staden J. (1994). Commercial Seaweed Products as Biostimulants in Horticulture. J. Home Consumer Horticult..

[57] Gharib F., Zeid I.M., Salem O., Ahmed E.Z. (2014). Effects of Sargassum Latifolium Extract on Growth, Oil Content and Enzymatic Activities of Rosemary Plants under Salinity Stress. Life Sci. J..

[58] Minhas P.S., Ramos T.B., Ben-Gal A., Pereira L.S. (2020). Coping with Salinity in Irrigated Agriculture: Crop Evapotranspiration and Water Management Issues. Agric. Water Manag..

[59] Bonomelli C., Celis V., Lombardi G., Mártiz J. (2018). Salt Stress Effects on Avocado (*Persea americana* Mill.) Plants with and without Seaweed Extract (*Ascophyllum nodosum*) Application. Agronomy.

[60] Hernández-Herrera R.M., Santacruz-Ruvalcaba F., Briceño-Domínguez D.R., Filippo-Herrera D., Andrea D., Hernández-Carmona G. (2018). Seaweed as Potential Plant Growth Stimulants for Agriculture in Mexico. Hidrobiológica.

[61] Sohn S.-I., Rathinapriya P., Balaji S., Jaya Balan D., Swetha T.K., Durgadevi R., Alagulakshmi S., Singaraj P., Pandian S. (2021). Phytosterols in Seaweeds: An Overview on Biosynthesis to Biomedical Applications. Int. J. Mol. Sci..

[62] Chanthini K.M.-P., Pavithra G.-S., Senthil-Nathan S., Malafaia G. (2024). An In-Depth Review on the Mechanistic Insights of Marine Macroalgal Compounds in Enhancing Plant Tolerance to Stress Induced by Saline Soil Conditions. Toxin. Rev..

[63] Bayomy H.M., Alamri E.S., Alharbi B.M., Foudah S.H., Genaidy E.A., Atteya A.K. (2023). Response of *Moringa oleifera* Trees to Salinity Stress Conditions in Tabuk Region, Kingdom of Saudi Arabia. Saudi J. Biol. Sci..

[64] Golestani Araghi S., Assad M.T. (1998). Evaluation of Four Screening Techniques for Drought Resistance and Their Relationship to Yield Reduction Ratio in Wheat. Euphytica.

[65] Chanthini K.M.-P., Senthil-Nathan S., Pavithra G.-S., Malarvizhi P., Murugan P., Deva-Andrews A., Janaki M., Sivanesh H., Ramasubramanian R., Stanley-Raja V. (2022). Aqueous Seaweed Extract Alleviates Salinity-Induced Toxicities in Rice Plants (*Oryza sativa* L.) by Modulating Their Physiology and Biochemistry. Agriculture.

[66] Osman H.E., Salem O. (2011). Effect of Seaweed Extracts as Foliar Spray on Sunflower Yield and Oil Content. Egypt. J. Phycol..

[67] Irving D.W., Shannon M.C., Breda V.A., Mackey B.E. (1988). Salinity Effects on Yield and Oil Quality of High-Linoleate and High-Oleate Cultivars of Safflower. J. Agric. Food Chem..

[68] Smirnoff N. (1993). Tansley Review No. 52. The Role of Active Oxygen in the Response of Plants to Water Deficit and Desiccation. New Phytol..

[69] Yeilaghi H., Arzani A., Ghaderian M., Fotovat R., Feizi M., Pourdad S.S. (2012). Effect of Salinity on Seed Oil Content and Fatty Acid Composition of Safflower (*Carthamus tinctorius* L.) Genotypes. Food Chem..

[70] Azachi M., Sadka A., Fisher M., Goldshlag P., Gokhman I., Zamir A. (2002). Salt Induction of Fatty Acid Elongase and Membrane Lipid Modifications in the Extreme Halotolerant Alga Dunaliella Salina. Plant Physiol..

[71] Hajlaoui H., Denden M., El Ayeb N. (2009). Changes in Fatty Acids Composition, Hydrogen Peroxide Generation and Lipid Peroxidation of Salt-Stressed Corn (*Zea mays* L.) Roots. Acta Physiol. Plant.

[72] Upchurch R.G. (2008). Fatty Acid Unsaturation, Mobilization, and Regulation in the Response of Plants to Stress. Biotechnol. Lett..

[73] Zhao F.-G., Qin P. (2005). Protective Effects of Exogenous Fatty Acids on Root Tonoplast Function against Salt Stress in Barley Seedlings. Environ. Exp. Bot..

[74] Konova I.V., Sergeeva Y.E., Galanina L.A., Kochkina G.A., Ivanushkina N.E., Ozerskaya S.M. (2009). Lipid Synthesis by Geomyces Pannorum under the Impact of Stress Factors. Microbiology.

[75] Elansary H.O., Skalicka-Woźniak K., King I.W. (2016). Enhancing Stress Growth Traits as Well as Phytochemical and Antioxidant Contents of Spiraea and Pittosporum under Seaweed Extract Treatments. Plant Physiol. Biochem..

[76] Isah T. (2019). Stress and Defense Responses in Plant Secondary Metabolites Production. Biol. Res..

[77] Zhang L., Becker D.F. (2015). Connecting Proline Metabolism and Signaling Pathways in Plant Senescence. Front Plant Sci..

[78] Mansour M.M.F., Ali E.F. (2017). Evaluation of Proline Functions in Saline Conditions. Phytochemistry.

[79] Acosta-Motos J.R., Ortuño M.F., Bernal-Vicente A., Diaz-Vivancos P., Sanchez-Blanco M.J., Hernandez J.A. (2017). Plant Responses to Salt Stress: Adaptive Mechanisms. Agronomy.

[80] Jaarsma R., de Vries R.S.M., de Boer A.H. (2013). Effect of Salt Stress on Growth, Na+ Accumulation and Proline Metabolism in Potato (*Solanum tuberosum*) Cultivars. PLoS ONE.

[81] Sarabi B., Bolandnazar S., Ghaderi N., Ghashghaie J. (2017). Genotypic Differences in Physiological and Biochemical Responses to Salinity Stress in Melon (*Cucumis melo* L.) Plants: Prospects for Selection of Salt Tolerant Landraces. Plant Physiol. Biochem..

[82] De la Torre-González A., Montesinos-Pereira D., Blasco B., Ruiz J.M. (2018). Influence of the Proline Metabolism and Glycine Betaine on Tolerance to Salt Stress in Tomato (*Solanum lycopersicum* L.) Commercial Genotypes. J. Plant Physiol..

